# Huge, Infected Pancreatic Necrosis After Total Arch Replacement in a Patient With Immunoglobulin G4-Related Syndrome

**DOI:** 10.7759/cureus.56805

**Published:** 2024-03-24

**Authors:** Tomohiro Nakajima, Yutaka Iba, Tsuyoshi Shibata, Ayaka Arihara, Nobuyoshi Kawaharada

**Affiliations:** 1 Cardiovascular Surgery, Sapporo Medical University, Sapporo, JPN

**Keywords:** steroid use, aneurysm, igg4-related syndrome, pancreatic necrosis, total arch replacement

## Abstract

A 77-year-old male patient with immunoglobulin (Ig)G4-related disease was diagnosed with a 60-mm aortic arch aneurysm and atherosclerosis of the aorta advanced throughout the body. Aortic arch replacement surgery was performed with circulatory arrest at 28°C. One week later, the patient developed acute pancreatitis, followed by encapsulated necrosis in the chronic phase. After debridement surgery, the patient’s condition improved.

## Introduction

Acute pancreatitis is morphologically classified into interstitial edematous pancreatitis, which is characterized by interstitial edema, and necrotizing pancreatitis, which involves extensive fat necrosis and parenchymal necrosis, both intra- and extrapancreatically. Approximately 80-90% of pancreatitis cases are edematous and may resolve without requiring specialized treatment, whereas 10-20% of cases are necrotizing pancreatitis, with a mortality rate of 14-25% [1].

## Case presentation

**Figure 1:**
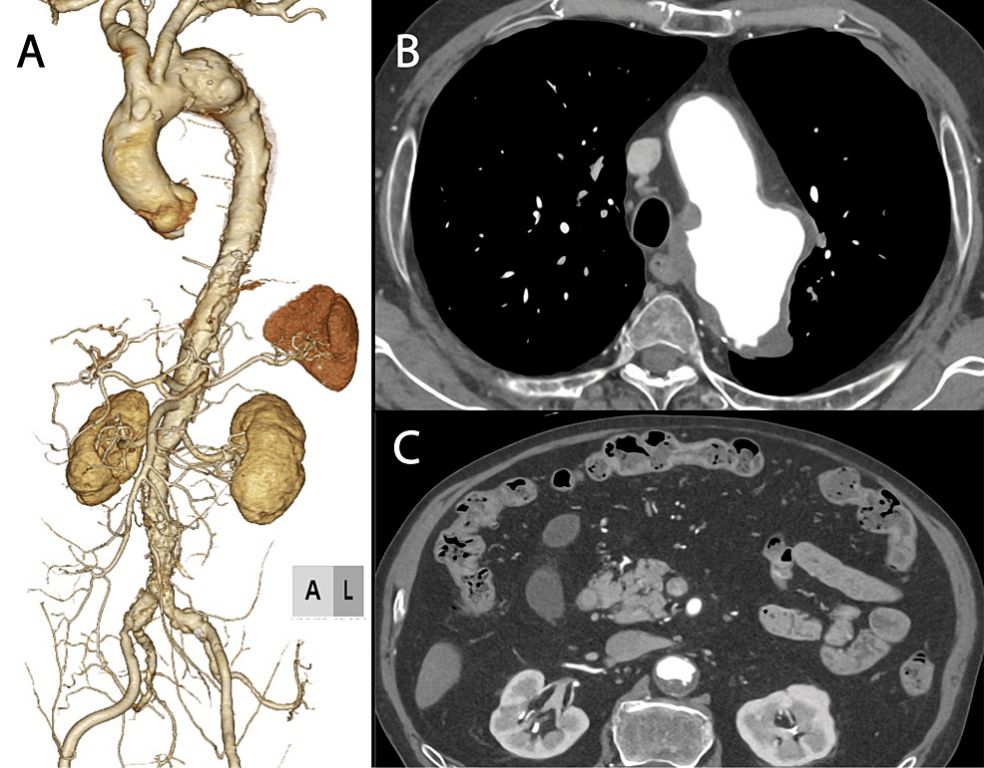
Computed tomography imaging before operation (A) Volume rendering image. The aortic arch aneurysm diameter was 60 mm. (B) Axial image at the aortic arch showed high atherosclerosis changes. (C) Axial image at the pancreas level indicated highly atherosclerotic changes in the aorta and normal pancreas.

A 77-year-old male patient, who was on oral administration of 40 mg hydrocortisone for IgG4-related disease was found to have a 60-mm aortic arch aneurysm on contrast-enhanced computed tomography (CT), which warranted surgical intervention (Figure 1A). CT revealed severe atherosclerosis throughout the aorta (Figures 1B, 1C).

**Figure 2:**
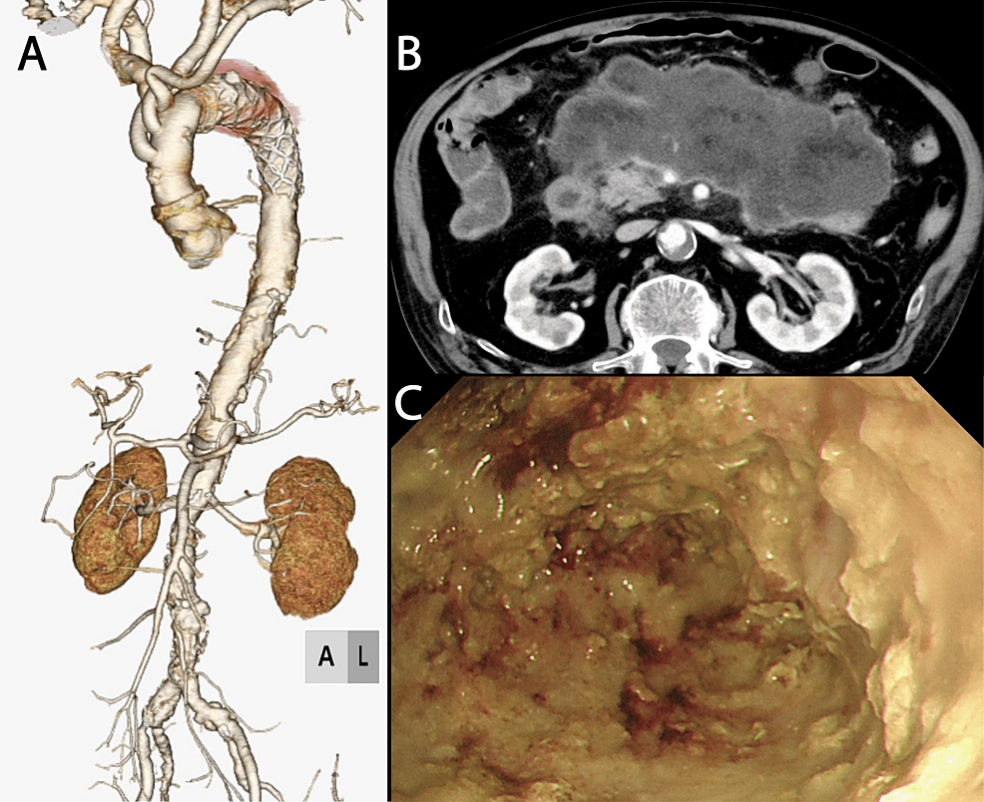
Computed tomography imaging after operation. (A) Volume rendering image. The aortic arch aneurysm was resected and the frozen elephant trunk was placed at the aortic arch and descending aorta. (B) An axial image at the pancreas level indicated a huge, infected pancreatic necrosis. (C) An image of the necrotic pancreas being debrided via gastrointestinal endoscopy. This is an image of the pancreas. Extensive necrotic tissue has been removed, exposing a pancreas that appears close to normal.

A brain isolation technique was employed to prevent embolization of the atheromatous plaque from the aortic arch. The procedure was initiated under general anesthesia. A 9-mm artificial graft was sutured to both axillary arteries. Subsequently, a median sternotomy was performed, a 24Fr cannula was inserted into the superior vena cava and a 28Fr cannula into the inferior vena cava. An 18Fr cannula was inserted into the ascending aorta. Cerebral perfusion was initiated through the bilateral axillary arteries, whereas antegrade perfusion from the ascending aorta was established to initiate cardiopulmonary bypass. Occlusion of the brachiocephalic trunk, left common carotid artery, and left subclavian artery was performed. A 12Fr catheter was inserted into the left common carotid artery through a semi-circumferential incision to facilitate brain isolation. The flow rate was set at 1000 mL/min and systemic hypothermia was achieved by additional antegrade perfusion from the left femoral artery. The circulatory arrest was initiated at a rectal temperature of 28°C. The left subclavian artery origin was closed, and a 30-mm 12-cm frozen elephant trunk (J graft Frozenix®, Japan LifeLine, Tokyo) was inserted. Rewarming was initiated after the left subclavian reconstruction. Central anastomosis was performed, followed by reconstruction of the left common carotid and brachiocephalic arteries. The total duration of circulatory arrest was 58 min, the aortic clamping time was 111 min, the cardiopulmonary bypass time was 255 min, and the surgical duration was 491 min. The postoperative course was uneventful. Postoperative contrast-enhanced CT revealed no abnormalities in the replaced aorta (Figure 2A). On postoperative day 11, acute pancreatitis developed, which was successfully managed conservatively by gastroenterologists. However, on postoperative day 20, the patient developed a fever of 39°C. Subsequent CT imaging revealed chronic pancreatitis with pancreatic cyst formation and signs of infection (Figure 2B). Using upper gastrointestinal endoscopy, gastroenterologists performed endoscopic debridement of the necrotic pancreatic cyst from the Vater papilla (Figure 2C).

Curettage of necrotic material was performed for seven consecutive days using endoscopy. The antibiotic used was tazobactam piperacillin. Thereafter, continuous irrigation (40 mL/h) was maintained for four weeks for the necrotic pancreatic cysts. While the pancreatic cyst infection was resolved, the patient developed respiratory failure pneumonia, necessitating a tracheostomy. The patient was transferred to another facility on postoperative day 120.

## Discussion

Aortic arch aneurysms account for approximately 50% of true thoracic aortic aneurysms [2]. They predominantly affect elderly individuals and are often associated with arteriosclerosis. Given the risk of cerebral embolism from arteriosclerotic plaques, the entire aortic arch was replaced with artificial blood vessels. Advancements in surgical techniques have improved patient outcomes by ensuring the implementation of reliable cerebral protection methods. With elective procedures, the mortality rate has decreased to approximately 5%, and acute pancreatitis has been reported to occur in 4% of cases. Encapsulated liquefied necrotic pancreatic or peripancreatic tissue is referred to as "walled-off necrosis” (WON). The mortality rate associated with this condition is extremely high, ranging from 30% to 40% [3]. Fortunately, in this case, we prevented mortality by transferring the patient to a different facility.

There are two possible causes of pancreatitis in this case. One is the impairment of pancreatic microcirculation due to pancreatic ischemia caused by circulatory arrest and hypothermia [4]. The second is the influence of pancreatic ischemia due to the impairment of pancreatic microcirculation by microembolism in patients with severe atherosclerosis [5]. To avoid pancreatitis in this case, the only option was to observe without surgery. However, it is unclear whether we were able to avoid aneurysm rupture through a natural course.

## Conclusions

A 77-year-old patient with IgG4-related disease on steroids underwent arterial aortic artery replacement after a circulatory arrest for an aortic arch aneurysm. Preoperatively, atherosclerosis was very severe. Postoperatively, the patient developed acute pancreatitis, which subsequently became chronic, and infection adhered to the pancreatic cysts. The infection was controlled by drainage with debridement. In this case, we believe that there were no specific measures taken to prevent pancreatitis. We consider early detection and appropriate treatment to be crucial.
